# Orofacial myofunctional and polysomnographic characteristics of children with Down syndrome and obstructive sleep apnea: a pilot study

**DOI:** 10.1590/2317-1782/20242023119en

**Published:** 2024-05-27

**Authors:** Danielle Barreto e Silva, Camila de Castro Corrêa, Silke Anna Theresa Weber

**Affiliations:** 1 Programa de Pós-graduação em Cirurgia e Medicina Translacional (Doutorado), Hospital das Clínicas, Faculdade de Medicina de Botucatu, Universidade Estadual Paulista – UNESP - Botucatu (SP), Brasil.

**Keywords:** Sleep Apnea, Obstructive, Child, Down Syndrome, Polysomnography, Speech, Language and Hearing Sciences, Stomatognathic System

## Abstract

**Purpose:**

To investigate oropharyngeal structures and functions in a pediatric population with Down Syndrome (DS) and obstructive sleep apnea (OSA) and to correlate with the apnea/hypopnea index (AHI) and sleep questionnaires.

**Methods:**

12 Children with DS and OSA, between the age of 4 and 12 years old, underwent polysomnography (PSG); sleep questionnaires, Pediatric Sleep Questionnaire (PSQ) and Obstructive Sleep Apnea-18 (OSA-18); and speech-language evaluation using the Short Evaluation of Orofacial Myofunctional Protocol (ShOM).

**Results:**

There was a positive correlation between ShoM higher scores and the apnea-hypopnea index (AHI) and between ShoM and the number of hypopneas. The orofacial myofunctional alterations observed in the studied group were: oral breathing, alteration in lip tonus and competence, tongue posture at rest and in swallowing, and occlusal alteration. There was also an increased risk for OSA according to the sleep questionnaires, as well as the presence of obesity and overweight, but without correlation with the severity of OSA.

**Conclusion:**

All DS children show alterations in orofacial characteristics, higher scores being associated to severe OSA. Orofacial myofunctional evaluation may help to identify different phenotypes in Down syndrome children with Obstructive sleep Apnea, enhancing the need for a multidisciplinary approach.

## INTRODUCTION

Down Syndrome (DS), or T21 (trisomy 21) is among the most prevalent genetic conditions in the world population. Its incidence is estimated at 1 case in 800 to 1792 live births^(1,2)^. Individuals with DS have phenotypic characteristics related to more than 200 protein-coding genes on chromosome 21 (HSA21-Homo sapiens chromosome 21), acting directly or indirectly on cellular homeostasis in tissues, organs, and systems^(3)^.

Craniofacial characteristics, muscle hypotonia, orofacial myofunctional disorders (OMD), metabolic changes, hypothyroidism, and a greater tendency to obesity in DS are important risk factors for the development of obstructive sleep apnea (OSA)^(3,4)^, which is chronic progressive sleep-disordered breathing characterized by total (apnea) or partial (hypopnea) obstruction of the upper airway (UA). Obstructive sleep apneas/hypopneas are intermittent and recurrent during sleep, accompanied by drops in oxyhemoglobin saturation and brief electroencephalographic awakenings that fragment sleep, causing metabolic, hormonal, cardiovascular, cognitive, and behavioral changes^(5,6)^.

OSA prevalence is estimated at 1 to 4% among 2-to-8-year-old children^(7)^, especially associated with UA obstruction and obesity^(8)^. This figure can increase to 13% to 59% in obese children due to fat accumulated in the UA^(9)^. The prevalence among children with DS ranges from 31% to 72%^(4,10,11)^, which demonstrates that OSA is significantly more recurrent in DS than in those without the genetic condition.

The UA configuration associated with mandibular and midface hypoplasia, malocclusions, skeletal class III, crossbite, overbite, dental crowding, and orofacial and cervical muscle hypotonia is related to the higher prevalence of OSA in DS^(4)^. However, studies demonstrate that orofacial myofunctional disorders such as reduced orofacial muscle tone, masticatory and swallowing muscle coordination, and mouth or oronasal breathing are frequent findings in the general population with OSA, even without T21^(12-14)^. Although such disorders are frequent findings in individuals with OSA, they can be more severe in people with DS due to the association between changes in craniofacial development, UA morphology, and global hypotonia^(4,12-14)^.

The frequent association between the two conditions, DS and OSA, and the high presence of comorbidities corroborate the importance of diagnosis and assertiveness in treatment^(4)^. However, using polysomnography (PSG) (the standard test for diagnosing sleep disorders) to identify OSA early in individuals with DS, especially children, is hampered by its high cost, difficulty accessing services that offer the exam, and specific criteria for the equipment and teams specialized in monitoring children^(4,7)^.

Screening questionnaires, scales, and protocols are low-cost, easy, and quick-to-apply instruments that help investigate sleep and the risk of disorders. The Pediatric Sleep Questionnaire (PSQ)^(15)^, the Obstructive Sleep Apnea-18 (OSA-18)^(16,17)^, (related to the quality of a child's sleep), and the Short Evaluation of Orofacial Myofunctional Protocol (ShOM) (a speech-language-hearing screening protocol to identify orofacial myofunctional disorders in individuals with OSA) seek to analyze the presence, frequency, and physical and behavioral consequences of symptoms in individuals at risk of OSA.

No study has analyzed these protocols in children with DS and their association with PSG data to date. It is hypothesized that identifying the craniofacial and muscle conditions of children with DS could help identify the risk and severity of OSA. Hence, this study aimed to investigate orofacial structures and functions in children with DS, correlating them with the presence and severity of OSA and the results of the PSQ and OSA-18 sleep questionnaires.

## METHOD

The Research Ethics Committee of the Medical School of Botucatu (FMB-UNESP) approved the research under CAAE protocol number 47939721.8.0000.5411.

The sample was recruited by convenience, comprising patients from the Sleep Disorders Outpatient Clinic at the Clinics Hospital of FMB-UNESP/Botucatu. The recruited group underwent previous medical consultation and assessment as part of the service routine to collect anthropometric data and apply the PSQ and OSA-18 questionnaires to investigate sleep. After signing an informed consent form, their orofacial myofunctional conditions were screened by a speech-language-hearing pathologist using the ShOM. The sample had 12 children with DS and OSA. All children underwent a sleep study at the Clinics Hospital of FMB-UNESP/Botucatu with type-III polygraph, manufactured by Philips, and the following components: airflow cannula and thermistor, chest and abdomen strap, pulse oximetry, and position sensor.

### Inclusion criteria

Children with DS and OSA, aged 4 to 12 years old, monitored at the Sleep Disorders Outpatient Clinic at the Clinics Hospital of FMB-UNESP/Botucatu, with PSG results, after signing an informed consent form.

### Exclusion criteria

Children with neurological disorders, neuromuscular disorders, or taking drugs that depress the respiratory system, according to medical examination and history.

OSA was diagnosed based on the apnea/hypopnea index (AHI), classifying OSA in children as a) mild OSA: AHI – 1.1 to 5 events/hour of sleep (e/h); b) Moderate OSA: AHI – 5.1 to 10 e/h; c) Severe OSA: IAH – above 10 e/h^(18)^.

PSQ and OSA-18 were used to assess sleep subjectively.

**PSQ** – intended for children aged 2 to 18 years, it has 22 items distributed in 3 domains that address the frequency of snoring, loud snoring, observed apneas, difficulty breathing during sleep, daytime sleepiness, inattentive or hyperactive behavior, and other pediatric characteristics of OSA. The score is defined by the mean response to non-missing items, ranging from 0 to 1. Scores above 7 indicate the presence of sleep-disordered breathing^(15,19)^
**OSA-18 (Portuguese version) –** is a questionnaire with 18 items grouped into five domains: a) sleep disturbance, b) distress, c) emotional distress, d) daytime problems, and e) concerns of parents/guardians. Items are scored on a scale of 1 to 7 points. The OSA-18 score ranges from 18 to 126 points and is categorized into three groups, according to the impact on quality of life: small – less than 60; moderate – from 60 to 80; and great – above 80. The more frequent the item in each domain, the higher the final score and the worse the impact on quality of life^(16,17)^.

Orofacial myofunctional disorders were screened with the ShOM, a protocol developed for such conditions in children with OSA. It was originally structured considering breathing mode and type, lip competence, lip tone, tongue posture at rest and swallowing, dilator naris tone, dental occlusion, Glatzel test, and Rosenthal test, totaling 10 items. The scores range from 0 (normal) to 1 (abnormal) – a sum of 10 means the greatest number of orofacial myofunctional changes possible in this protocol^(20)^. The protocol was adapted to the selected population, using 7 of the 10 items, excluding the assessment of breathing type, dilator naris tone, and Glatzel test.

The speech-language-hearing screening analyzed videos recorded during consultations at the Sleep Disorders Outpatient Clinic at the Clinics Hospital of FMB-UNESP/Botucatu, upon specific authorization in the informed consent form. They were recorded by a person trained by the responsible speech-language-hearing pathologists based on a pre-established process to enable the assessment of each ShOM item. The recordings were then separately analyzed by two speech-language-hearing pathologists with experience in oral motor therapy, according to criteria already established in speech-language-hearing clinical practice. The screening was carried out without prior knowledge of the PSG and sleep questionnaire results.

### Data analysis

The descriptive analysis presented data in frequency, mean, and standard deviation. Data from PSQ, OSA-18, and ShOM were compared with each other and those on PSG, AHI, obstructive apnea, hypopnea, central apneas, and oxyhemoglobin saturation, using the Jamovi program (version 1.2.2.5) and Spearman correlation test. The significance level was set at < 0.05 (*).

## RESULTS

The group had 12 children with DS and OSA, of which six were males, with a mean age of 8.08 years (± 2.75), a minimum age of 4 years, and a maximum age of 12 years. Data on the individual variables analyzed are described in Table 1.

**Table 1:** Individual data from children with Down syndrome and obstructive sleep apnea

N	Age	Sex	BMI	Z-score	AHI	OA	CA	HYPO	Min SpO_2%_	Mean SpO_2%_	SpO_2%_ < 90	PSQ	OSA-18	ShOM
1	7	F	**27.3**	obesity	1.7	1.2	0.3	0.2	92	97	0.0	14	94	4
2	10	M	**27.3**	obesity	6.3	0.6	1.8	3.9	81	93	9.3	8	67	5
3	9	F	**20.5**	overweight	6.8	1.4	3.7	1.7	90	97	0.0	3	31	6
4	10	F	**18.4**	normal weight	8.4	3.1	4.3	0.9	89	98	0	11	100	2
5	5	F	**15.4**	normal weight	8.6	0	2.5	6.1	87	95	0.1	6	45	5
6	11	M	**26**	obesity	9.7	0.7	1.8	7.1	85	96	0.1	7	38	6
7	7	M	**24**	obesity	11	4.3	0.2	6.5	88	97	0.0	9	84	7
8	12	F	**29**	obesity	11.2	11.2	3	0	74	94	4.7	2	41	5
9	11	M	**16.2**	normal weight	18.6	0.1	4	14.5	89	96	0.0	8	60	6
10	5	F	20.4	overweight	20.4	2.8	0	17.6	82	95	0.2	18	107	7
11	4	M	**17.2**	overweight	21.8	11.6	0	10.1	78	94	5.1	9	38	6
12	6	M	**23.9**	obesity	25.1	0.9	6.7	17.5	76	96	1.0	20	97	6

Caption: N = individual; BMI = body mass index; AHI = apnea/hypopnea index; OA = obstructive apnea; CA = central apnea; HYPO = hypopnea; Min SpO_2%_ = minimum oxygen saturation percentage; Mean SpO_2%_ = mean oxygen saturation percentage; SpO_2%_ < 90 = percentage of time with oxygen saturation below 90; PSQ = Pediatric Sleep Questionnaire; OSA-18 = OSA-18 Protocol; ShOM = Short Evaluation of Orofacial Myofunctional Protocol

Nutritional data used the criteria of body mass index (BMI = weight [kg]/height [m^2^]), sex, and age. Cutoff points form BMI-for-age scores in children (Z-score)^(21)^; six children were found to be obese, and three were overweight (Table 1).

The orofacial myofunctional screening analyzed seven out of the 10 ShOM items through recordings made during consultations at the Sleep Disorders Outpatient Clinic at the Clinics Hospital of FMB-UNESP/Botucatu. The following items were assessed: breathing mode, lip competence, lip tone, tongue position, tongue position during swallowing, dental occlusion, and Rosenthal Test, as described in Table 2. The group mean score was 5.42 (±1.4), with a minimum of 4 and a maximum of 7. All individuals had orofacial myofunctional changes. The higher the score, the greater the orofacial myofunctional impairment (Tables 1 and 2).

**Table 2:** Results of the items assessed with the ShOM protocol

ShOM	DS (N = 12)	
Maximum score = 7	Abnormal	Normal
Breathing mode	10 (83.33%)	2 (16.66%)
Lip competence	6 (50%)	6 (50%)
Lip tone	10 (83.33%)	2 (16.66%)
Tongue position	12 (100%)	0
Tongue position in swallowing	12 (100%)	0
Occlusion	5 (41.66%)	7 (58.33%)
Rosenthal test	10 (83.33%)	2 (16.66%)

Caption: ShOM = Short Evaluation of Orofacial Myofunctional Protocol; DS = Down syndrome; N = number of individuals

As for PSG data, the mean AHI was 12.5 e/h of sleep (±7.24) – one child (8.33%) was diagnosed with mild OSA (1.1 – 5 e/h), five children (41.66%) with moderate OSA (5.1 – 10 e/h), and six children (50%) with severe OSA (> 10 e/h) (Table 3). In addition to obstructive apnea and hypopnea, central apnea was also found, in which respiratory effort is absent or reduced, with a mean of 2.36 e/h of sleep (± 2.09) – which may be related to other health conditions such as cardiovascular changes, common comorbidities in this population. The mean minimum oxygen saturation percentage (SpO_2_) was 84.3 (± 5.94), the mean SpO_2_ was 95.7 (±1.5), and the percentage of time below 90% SpO_2_ was 1.71 (±3.02) (Table 3).

**Table 3:** Correlation between ShOM and AHI and between ShOM and hypopnea

Spearman’s correlation*																				
M ± SD			AGE	BMI	AHI		OA	CA	MA	HYPO		Min SpO_2%_		Mean SpO_2%_		SpO_2%_ < 90	PSQ		OSA-18	ShOM
AGE	8.08 (± 2.75)	Spearman's rho	—																	
		p value	—																	
BMI	22.1 (± 4.73)	Spearman's rho	0.462	—																
		p value	0.13	—																
AHI	12.5 (± 7.24)	Spearman's rho	-0.289	-0.319	—															
		p value	0.363	0.313	—															
OA	3.16 (± 4.06)	Spearman's rho	-0.13	0.207	0.273		—													
		p value	0.687	0.519	0.391		—													
CA	2.36 (± 2.09)	Spearman's rho	0.454	-0.118	-0.018		-0.333	—												
		p value	0.138	0.716	0.957		0.29	—												
MA	0.01 (± 0.034)	Spearman´s rho	-0.098	-0.065	0.259		0.194	-0.39	—											
		p value	0.762	0.841	0.416		0.545	0.21	—											
HYPO	7.17 (± 6.46)	Spearman's rho	-0.44	-0.473	0.734	^**^	-0.189	-0.168	0.259	—										
		p value	0.152	0.121	0.007		0.557	0.601	0.416	—										
Min SpO_2%_	84.3 (±5.94)	Spearman's rho	0.074	-0.218	-0.62	^*^	-0.235	0.081	-0.26	-0.277		—								
		p value	0.819	0.497	0.032		0.463	0.803	0.415	0.384		—								
Mean SpO_2%_	95.7 (± 1.5)	Spearman's rho	0.097	-0.093	-0.306		0.064	0.321	-0.231	-0.192		0.767	**	—						
		p value	0.765	0.774	0.333		0.843	0.308	0.471	0.55		0.004		—						
SpO_2%_ < 90	1.71 (± 3.02)	Spearman's rho	-0.165	0.229	0.36		0.105	-0.235	0.303	0.189		-0.899	^***^	-0.9	***	—				
		p value	0.609	0.474	0.251		0.745	0.462	0.339	0.557		< .001		< .001		—				
PSQ	10.1 (± 4.94	Spearman's rho	-0.355	0.132	0.396		0.396	-0.116	-0.163	0.263		-0.213		0.138		0.102	—			
		p value	0.257	0.683	0.202		0.202	0.719	0.614	0.409		0.507		0.67		0.752	—			
OSA-18	66.8 (± 28.3)	Spearman's rho	-0.22	0	0.088		0.007	0.028	-0.519	0.207		0.049		0.244		-0.115	0.8	**	—	
		p value	0.491	1	0.787		0.983	0.931	0.084	0.519		0.879		0.444		0.723	0.002		—	
ShOM	5.42 (± 1.38)	Spearman's rho	-0.291	-0.165	0.604	*	0.154	-0.369	0.203	0.75	**	-0.176		-0.061		0.019	0.064		-0.059	—
		p value	0.358	0.608	0.038		0.633	0.238	0.526	0.005		0.584		0.849		0.953	0.843		0.856	—


* p < .05;

** p < .01;

*** p < .001

Caption: M = mean; SD = standard deviation; BMI = body mass index; AHI = apnea/hypopnea index; OA = obstructive apnea; CA = central apnea; MA = mixed apnea; HYPO = hypopnea; Min SpO_2%_ = minimum oxygen saturation percentage; Mean SpO_2%_ = mean oxygen saturation percentage; SpO_2%_ < 90 = percentage of time with oxygen saturation below 90; PSQ = Pediatric Sleep Questionnaire; OSA-18 = OSA-18 Protocol; ShOM = Short Evaluation of Orofacial Myofunctional Protocol

The mean scores in the sleep-related questionnaires were 10.1 (± 4.94) in PSQ and 66.8 (± 28.3) in OSA-18. The scores obtained from both questionnaires suggest an increased risk for OSA (Table 3).

The Spearman's correlation coefficient test verified that ShOM was positively correlated with AHI (p = 0.038) and the number of hypopneas (p = 0.005), as shown in Table 3. These correlations show that higher ShOM scores are related to greater severity of OSA.

## DISCUSSION

OSA affects 31% to 71% of children with DS^(4,10,11)^, while the prevalence in typically developing children is 1% to 4%^(7)^. The increased risk for OSA in children with DS is mainly due to phenotypes related to hypotonia and craniofacial characteristics such as midface and cranial base hypoplasia.

The muscle condition and orofacial and pharyngeal functions in DS contribute to the complete or partial UA collapse during obstructive sleep apnea and/or hypopnea, thus influencing the AHI, one of the OSA severity parameters^(22)^. The results showed that all children in this study with DS and OSA had orofacial myofunctional changes, with varying levels of oral structure impairment in terms of lip and tongue posture and tone and abnormal breathing, mastication, and swallowing functions. Despite the small sample size, the ShOM score was positively correlated with AHI (p = 0.038) and the number of hypopneas (p = 0.005), thus suggesting that the higher the ShOM score (i.e., the greater the orofacial myofunctional impairment), the greater the severity of OSA.

The identification of clinical markers with the potential to predict OSA in children with DS may facilitate early diagnosis and treatment and the prevention of morbidities. However, to date, the literature presents an inconsistent or weak association between clinical predictors and biomarkers for the presence of OSA in this population^(23)^.

The present study used seven of the original 10 ShOM items because the commands to perform the three excluded tests (breathing type, dilator naris tone, and Glatzel mirror) were difficult for study participants to understand and to analyze through videos alone. This protocol enables a quick and standardized assessment of frequent orofacial myofunctional changes in children with obstructive sleep-related respiratory disorders. However, it has not yet been validated, nor has a cutoff score been established as a parameter for identifying OSA. Further studies with larger samples should measure the potential and weight of each item separately.

Its mean overall score was 5.42 (±1.4), corresponding to 77% of the maximum score of 7, observing predominantly mouth or oronasal breathing and abnormal results in lip tone and competence, tongue posture at rest and during swallowing, dental occlusion, and the Rosenthal Test. The multicenter study by Corrêa et al. (2020)^(13)^ assessed Brazilian and Italian typical children with OSA using PSG, ShOM, OSA-18, and the Sleep Clinical Record – an Italian protocol that analyzes clinical parameters and symptoms to estimate the risk for OSA. They identified orofacial myofunctional changes similar to this pilot study, such as changes in lip tone, mouth breathing, dental malocclusion, changes in tongue posture at rest and swallowing, lip incompetence, and changes in the Rosenthal and Glatzel tests. The mean ShOM score was 5.64 ± 2.27, corresponding to 54% of the maximum score of 10. However, since it used the 10 original protocol items, typical children with OSA had lower scores than those with DS and OSA in this study. This suggests that the present study children had greater orofacial myofunctional impairments, which is compatible with the literature as they are children with DS^(4,6,11,23)^.

It has been difficult or impossible for studies to prove that factors such as age, sex, cigarette exposure, clinical findings, anthropometric data, and comorbidities (other than congestive heart disease) are predictive of the severity of OSA in children with DS^(23,24)^. On the other hand, the correlation presented in this pilot study between orofacial myofunctional changes and the severity of OSA had never been investigated or identified.

Studies indicate that the highest incidence of OSA in children occurs between 2 and 8 years old^(7)^. Diez et al. (2003) described male children under 8 years old with adenotonsillar hypertrophy as predictive factors for OSA^(24)^. The study group’s mean age was 8.08 years (± 2.75), encompassing six male and six female children; 25% of them were overweight, and 50% were obese, although with no statistical correlation with AHI. Dyken et al. (2003) stated that higher BMI was associated with severe OSA in older children^(25)^. Its correlation with overweight and obesity has already been proved in the adult population, but there is still controversy when it comes to children^(23)^. It must be considered that the study group had characteristics other than high BMI that influence the severity of the disorder, such as their genetic condition and the compromised morphology and functioning of oropharyngeal structures and respiratory tract^(12,23)^. A study by Hizal et al. (2022) found no correlations between OSA, age, BMI, and tonsil size – like the age and BMI findings in the present study^(25)^.

Even though OSA is very prevalent in DS, it is still underdiagnosed and presents a wide variety of comorbidities such as neurocognitive and learning disorders and behavioral, cardiovascular, and metabolic changes. The development of comorbidities may be associated with not only AHI and oxygen desaturation but also central apnea – as identified in this study, with a mean of 2.36 e/h of sleep (±2.09) –, thus interfering with child growth and development, and reinforcing the importance of early diagnosis^(24,25)^.

The American Academy of Pediatrics recommends investigating OSA in DS as early as 6 months old when there are complaints such as “heavy” breathing, snoring, unusual sleeping position, daytime sleepiness, respiratory arrest, and behavioral problems^(26)^. In the absence of complaints during routine pediatric visits, it is suggested that all children with DS undergo PSG (the gold standard test for diagnosing sleep disorders) between 3 and 4 years old^(11,24,26)^. However, children with DS still have restricted access to PSG even in academic settings, such as where this study was developed, and the test is applied later than recommended. This is due to the high cost of the exam and the few services with equipment and teams specialized in performing PSG in children^(4,7,15,27)^.

In addition to PSG, sleep screening instruments for children, such as the PSQ^(15)^ and OSA-18^(16,17)^, are part of the routine investigation of individuals with sleep disorders, helping to identify risk for the diagnosis of OSA. They are important tools if PSG cannot be performed, as they are easy and quick to apply and seek to analyze the presence, frequency, and physical and behavioral consequences of symptoms. However, these questionnaires have limited validation in children, as only 13% to 27% of parents/guardians recognize the existence of problems related to their children's sleep^(28)^.

The PSQ investigates different aspects of sleep quality, snoring, and behavioral issues such as inattention and hyperactivity. Its sensitivity and specificity have been evaluated respectively at 78% and 72%^(19)^, demonstrating that the assessment of sleep quality can be an important parameter in identifying children at potential risk for OSA^(29)^. The mean PSQ score in this study was 9.6 (± 5.5), showing an increased risk for OSA in children with DS, though with no correlation with the severity of OSA.

The OSA-18 reflects the impact of sleep on children's quality of life, considering physical and emotional suffering, daytime problems, and parental concerns. However, the study sample’s mean score of 66.8 (± 28.6) (i.e., a moderate impact on quality of life) was not correlated with AHI – confirming data already described in the guidelines of the European Respiratory Society Task Force, which suggests that the questionnaire has low sensitivity and specificity^(29,30)^. Moreover, orofacial myofunctional conditions were not correlated with the scores in the child sleep screening instrument.

The study by Corrêa et al. (2020) demonstrated a correlation between higher AHI and abnormal tongue posture at rest and in swallowing^(13)^ in typical children with OSA. This corroborates the results observed in this pilot study, which found a relationship between postural and functional changes in oropharyngeal structures and a greater number of obstructive sleep apnea/hypopneas.

Although the small sample size is a limitation in the results of this pilot study, it confirmed the hypothesis that identifying orofacial myofunctional conditions in children with DS can help identify the risk and severity of OSA. Thus, considering that orofacial myofunctional disorders are present to a lesser or greater extent in individuals with DS, it is essential to identify and quantify changes in orofacial muscle posture, mobility, and tone and breathing, sucking, swallowing, mastication, and speech functions – which calls for the inclusion of speech-language-hearing pathologists in multidisciplinary teams of sleep specialists^(4,6,11-13)^.

This is a pilot study. Hence, further research with populations determined by sample size calculation is needed to understand the relationship between orofacial myofunctional changes in children with DS and the presence and severity of OSA and validate the questionnaires used in this population.

Lastly, the search for auxiliary instruments and methods to diagnose obstructive sleep-disordered breathing in children aims to optimize the identification of risk and severity of OSA when PSG (the gold standard exam for diagnosing sleep disorders) is unfeasible or impossible. Assertive and early diagnosis can minimize comorbidities, objectively identifying obstructive and central breathing patterns during sleep that negatively impact the physical, cognitive, and behavioral health of children with DS and OSA.

## CONCLUSIONS

Orofacial myofunctional assessment can be considered an important resource for the clinical investigation of the risk for OSA in children with DS. The ShOM was positively correlated with the AHI and the number of hypopneas – hence, the greater its score (and, therefore, the worse the orofacial myofunctional condition), the greater the severity of OSA. Thus, it is important to include speech-language-hearing assessment as an integral part of the risk investigation for sleep-disordered breathing.
