# Supplementary material for: Orofacial myofunctional and polysomnographic characteristics of children with Down syndrome and obstructive sleep apnea: a pilot study
Source: Codas. 2024 May 27;36(3):e20230119. doi: 10.1590/2317-1782/20242023119en (PMC11166037; doi:10.1590/2317-1782/20242023119en)
Supplement: Translation

# Translation: Características miofuncionais orofaciais e polissonográficas de crianças com Síndrome de Down e apneia obstrutiva do sono: estudo piloto

**DOI:** 10.1590/2317-1782/20242023119pt

**Authors:** Danielle Barreto e Silva, Camila de Castro Corrêa, Silke Anna Theresa Weber

**Affiliations:** 1 Programa de Pós-graduação em Cirurgia e Medicina Translacional (Doutorado), Hospital das Clínicas, Faculdade de Medicina de Botucatu, Universidade Estadual Paulista – UNESP - Botucatu (SP), Brasil.

**Keywords:** Apneia Obstrutiva do Sono, Criança, Síndrome de Down, Polissonografia, Fonoaudiologia, Sistema Estomatognático

## Abstract

**Objetivo:**

Investigar as estruturas e funções orofaríngeas de uma população pediátrica com Síndrome de Down (SD) e apneia obstrutiva do sono (AOS) e correlacionar com o índice de apneia/hipopneia (IAH) e questionários do sono.

**Método:**

12 Crianças com SD e AOS, entre 4 e 12 anos, foram submetidas à polissonografia (PSG); questionários do sono, Pediatric Sleep Questionnaire (PSQ) e Obstructive Sleep Apnea-18 (OSA-18); e triagem fonoaudiológica por meio do Short Evaluation of Orofacial Myofunctional Protocol (ShOM).

**Resultados:**

Verificou-se uma correlação positiva entre pontuações mais elevadas no ShOM e o índice de apneia hipopneia (IAH) e entre o ShOM e número de hipopneias. As alterações miofuncionais orofaciais observadas no grupo estudado foram: respiração oral, alteração no tônus e competência labial, na postura de língua em repouso e na deglutição e alteração oclusal. Verificou-se também, um risco aumentado para AOS conforme os questionários do sono, bem como presença de obesidade e sobrepeso, mas sem correlação com a gravidade da AOS.

**Conclusão:**

Todas as crianças apresentaram alterações miofuncionais orofaciais, sendo que escores mais altos no ShOM, ou seja, um maior comprometimento miofuncional orofacial, estavam associados à maior gravidade de AOS, sugerindo que a avaliação miofuncional orofacial dentro de uma abordagem multidisciplinar pode auxiliar na identificação de fatores de risco para AOS em crianças com SD.

## INTRODUÇÃO

A Síndrome de Down (SD), ou T21 (Trissomia do 21) está entre as condições genéticas mais prevalentes na população mundial. Estima-se que a incidência é de 1 em 800 a 1792 casos de nascidos vivos^(1,2)^. Indivíduos com SD apresentam características fenotípicas relacionadas a mais de 200 genes codificadores de proteínas do cromossomo 21 (HSA21-*Homo sapiens chromosome* 21), com ação direta ou indireta na homeostase celular, nos tecidos, órgãos e sistemas^(3)^.

As características craniofaciais, hipotonia da musculatura, presença de distúrbios miofuncionais orofaciais (DMOs), alterações metabólicas, hipotiroidismo e uma maior tendência à obesidade na SD configuram importantes fatores de risco para o desenvolvimento da apneia obstrutiva do sono (AOS)^(3,4)^, um transtorno respiratório do sono crônico, progressivo, caracterizado por obstrução total (apneias) ou parciais (hipopneias) da via aérea superior (VAS). As apneias/hipopneias obstrutivas do sono são intermitentes e recorrentes durante o período de sono, sendo acompanhadas de quedas na saturação de oxihemoglobina com breves despertares eletroencefalográficos que fragmentam o sono, promovendo alterações metabólicas, hormonal, cardiovascular, cognitivo e comportamental^(5,6)^.

É estimada uma prevalência de AOS entre 1 a 4% das crianças com idade entre 2 e 8 anos^(7)^, principalmente associada a fatores obstrutivos em (VAS) e obesidade^(8)^. Em crianças obesas esse número pode aumentar para 13% a 59%, devido ao acúmulo de gordura na VAS^(9)^. Em crianças com SD a prevalência varia entre 31% a 72%^(4,10,11)^, evidenciando assim, um aumento significativo da AOS na SD em relação à população sem a condição genética.

A configuração da VAS associada à hipoplasia mandibular e de terço médio da face, maloclusões, classe III esquelética, mordida cruzada, sobremordida, apinhamentos dentários e hipotonia da musculatura orofacial e cervical está relacionada à maior prevalência da AOS na SD^(4)^. No entanto, estudos demonstram que distúrbios miofuncionais orofaciais (DMOs) como, tônus da musculatura orofacial rebaixado, reduzida coordenação dos músculos mastigatórios e da deglutição, e respiração oral (RO) ou mista são achados frequentes na população em geral que apresenta AOS, mesmo sem a T21^(12-14)^. Apesar das alterações miofuncionais orofaciais serem achados frequentes em indivíduos com AOS, na população com SD, os DMOs podem cursar em uma pior condição devido à associação entre as alterações do desenvolvimento craniofacial, morfologia da VAS e hipotonia global^(4,12-14)^.

A associação frequente entre as duas condições, SD e AOS, e a elevada presença de comorbidades corroboram a importância do diagnóstico e assertividade no tratamento^(4)^. Entretanto, a identificação precoce da AOS em indivíduos com SD, especialmente na infância, pela polissonografia (PSG), exame padrão para o diagnóstico dos distúrbios do sono, é prejudicada em razão do alto custo; dificuldade de acesso a serviços que ofereçam o exame; e critérios específicos em relação aos equipamentos e equipes especializadas na monitoração de crianças^(4,7)^.

Instrumentos de rastreio como questionários, escalas e protocolos são considerados de baixo custo, fácil e rápida aplicação, auxiliando na investigação do sono e risco de distúrbios. Os questionários *Pediatric Sleep Questionnaire* (PSQ)^(15)^ e *Obstructive Sleep Apnea-18* (OSA-18)^(16,17)^, relacionados à qualidade do sono da criança, assim como, o *Short Evaluation of Orofacial Myofunctional Protocolo (ShOM),* protocolo de triagem fonoaudiológica para identificação dos DMOs em indivíduos com AOS, buscam analisar a presença de sintomas, frequência, repercussões físicas e comportamentais em indivíduos com risco de AOS.

Até o momento, não há estudos que analisam os protocolos citados em crianças com SD e a associação com dados polissonográficos. Hipotetiza-se que a identificação das condições craniofaciais e musculares de crianças com SD poderiam auxiliar na identificação de risco e gravidade da AOS. Assim, a proposta deste estudo é investigar as estruturas e funções orofaciais em crianças com SD correlacionando-as com a presença e gravidade da AOS e com os resultados dos questionários do sono PSQ e OSA-18.

## MÉTODO

A pesquisa foi aprovada pelo Comitê de Ética em Pesquisa da Faculdade de Medicina de Botucatu/UNESP, sob o número do protocolo CAAE: 47939721.8.0000.5411.

A amostra selecionada é considerada uma amostra de conveniência composta por pacientes do Ambulatório dos Distúrbios do Sono do Hospital das Clínicas da FMB -UNESP/Botucatu. O grupo recrutado foi submetido à consulta e avaliação médica anterior, em que dados antropométricos e os questionários PSQ e OSA-18, relacionados à investigação do sono, foram coletados como parte da rotina do serviço. A triagem fonoaudiológica das condições miofuncionais orofaciais se deu por meio do protocolo ShOM após assinatura do Termo de Consentimento Livre e Esclarecido. A amostra foi composta de 12 crianças com SD e AOS. Todas as crianças realizaram estudo do sono no Hospital das Clínicas da FMB -UNESP/Botucatu, com o equipamento Polígrafo tipo III, marca Philips, componentes: cânula e termístor de fluxo aéreo, cinta tórax e abdômen, oximetria de pulso e sensor de posição

### Critério de inclusão

Crianças com SD e AOS, entre 4 e 12 anos acompanhadas no Ambulatório dos Distúrbios do Sono do Hospital das Clínicas da FMB -UNESP/Botucatu, com resultado de exame polissonográfico e após assinatura de Termo de Consentimento.

### Critério de exclusão

Crianças com distúrbios neurológicos, distúrbios neuromusculares ou uso de drogas que deprimem o sistema respiratório, conforme determinado por exame médico e anamnese.

O critério de diagnóstico de AOS foi realizado por meio do índice de apneia/hipopneia (IAH), classificando a AOS na criança como: a) AOS leve: IAH - 1.1 a 5 eventos/hora de sono (e/h); b) AOS moderada: IAH - 5.1 a 10 e/h; c) AOS gave: IAH – acima de 10 e/h^(18)^.

Para a avaliação subjetiva do sono, foram utilizados os questionários PSQ e OSA-18.

***Pediatric Sleep Questionnaire* (PSQ)** – direcionado a crianças de 2 a 18 anos. Contém 22 itens distribuídos em 3 domínios que questionam sobre a frequência do ronco, ronco alto, apneias observadas, dificuldade para respirar durante o sono, sonolência diurna, comportamento desatento ou hiperativo e outras características pediátricas da AOS. A resposta média nos itens não omissos é a pontuação, que pode variar de 0 a 1. A pontuação acima de 7 se relaciona à presença de um distúrbio respiratório do sono^(15,19)^
**OSA-18 (OSA-18- Versão portuguesa) -** consiste em um questionário de 18 itens agrupados em cinco domínios: a) distúrbio do sono, b) sofrimento, c) sofrimento emocional, d) problemas diurnos e e) preocupações dos pais ou responsáveis. Os itens são pontuados em escala de 1 a 7 pontos. A pontuação do OSA-18 pode variar de 18 a 126 pontos e são categorizados em três grupos, de acordo com o impacto na qualidade de vida: pequeno – menor que 60; moderado – entre 60 e 80; e grande – acima de 80; quanto mais frequente é o item em cada do domínio, maior a pontuação final e pior é o impacto na qualidade de vida^(16,17)^.

A triagem miofuncional orofacial foi realizada por meio do protocolo ***Short Evaluation of Orofacial Myofunctional Protocol*** (ShOM), um protocolo para Triagem Miofuncional Orofacial em crianças com AOS. Originalmente, foi estruturado considerando: modo e tipo respiratório, competência labial, tônus labial, postura da língua no repouso e na deglutição, tônus do dilatador da asa do nariz, oclusão dentária, teste de Glatzel e teste de Rosenthal, totalizando 10 itens. Foram pontuados de 0 (normal) a 1 (alteração); uma soma de 10 significa o maior número de alterações miofuncionais orofaciais possíveis dentro deste protocolo^(20)^. Foi realizada uma adaptação do protocolo para a população selecionada, sendo considerado 7 dos 10 itens, excluindo a avaliação do tipo respiratório, tônus do dilatador da asa do nariz e teste de *Glatzel.*

A triagem fonoaudiológica foi realizada por meio da análise de vídeos gravados durante as consultas no ambulatório dos Distúrbios do Sono do Hospital das Clínicas da FMB -UNESP/Botucatu, após autorização específica informada no Termo de Consentimento. A gravação foi realizada por uma pessoa treinada pelas fonoaudiólogas responsáveis a partir de um roteiro pré-estabelecido, de maneira que fosse possível avaliar cada item que compõe o protocolo ShOM. A análise das filmagens foi realizada posteriormente por duas fonoaudiólogas, separadamente, com experiência na especialidade de Motricidade Orofacial, de acordo com critérios já estabelecidos na clínica fonoaudiológica. A triagem foi realizada sem o conhecimento prévio dos resultados da polissonografia e dos questionários do sono.

### Análise dos dados

Foi realizada análise descritiva, apresentando a frequência, média e desvio padrão. Os dados dos questionários/protocolos PSQ, OSA-18 e ShOM foram comparados entre si e com os dados polissonográficos, índice de apneia e hipopneia (IAH), apneias obstrutivas, hipopneias, apneias centrais e saturação de oxihemoglobina, por meio do programa *Jamovi* (versão 1.2.2.5), utilizando o teste de correlação de *Spearman.* Foi adotado o nível de significância de < 0,05 (*).

## RESULTADOS

O grupo foi composto de 12 crianças com SD e AOS; sendo 6 do sexo masculino, com idade média de 8,08 anos (± 2,75), idade mínima de 4 anos e máxima de 12 anos. Os dados referentes a cada variável individual analisada estão descritos na Tabela 1.

**Tabela 1:** Dados individuais de crianças com SD e AOS

N	Idade	Sexo	IMC	Escore-z	IAH	AO	AC	HIPO	%SPO_2_ MIN	%SPO_2_ MÉD	%SPO_2_ <90	PSQ	OSA-18	ShOM
01	7	F	**27,3**	obesidade	1,7	1,2	0,3	0,2	92	97	0,0	14	94	4
02	10	M	**27,3**	obesidade	6,3	0,6	1,8	3,9	81	93	9,3	8	67	5
03	9	F	**20,5**	sobrepeso	6,8	1,4	3,7	1,7	90	97	0,0	3	31	6
04	10	F	**18,4**	eutrofia	8,4	3,1	4,3	0,9	89	98	0	11	100	2
05	5	F	**15,4**	eutrofia	8,6	0	2,5	6,1	87	95	0,1	6	45	5
06	11	M	**26**	obesidade	9,7	0,7	1,8	7,1	85	96	0,1	7	38	6
07	7	M	**24**	obesidade	11	4,3	0,2	6,5	88	97	0,0	9	84	7
08	12	F	**29**	obesidade	11,2	11,2	3	0	74	94	4,7	2	41	5
09	11	M	**16,2**	eutrofia	18,6	0,1	4	14,5	89	96	0,0	8	60	6
10	5	F	20,4	sobrepeso	20,4	2,8	0	17,6	82	95	0,2	18	107	7
11	4	M	**17,2**	sobrepeso	21,8	11,6	0	10,1	78	94	5,1	9	38	6
12	6	M	**23,9**	obesidade	25,1	0,9	6,7	17,5	76	96	1,0	20	97	6

Legenda: SD = Síndrome de Down; AOS = apneia obstrutiva do sono; N = indivíduo; IMC = índice de massa corpórea; IAH = índice de apneia e hipopneia; AO = apneia obstrutiva; AC = apneia central; HIPO = hipopneias; %SPO_2_ MIN = porcentagem de saturação de oxigênio mínima; %SPO_2_ MÉD = porcentagem de saturação de oxigênio média; %SPO_2_ <90 = porcentagem de tempo com saturação de oxigênio abaixo de 90; PSQ = *Pediatric Sleep Questionnaire*; OSA-18 = Protocolo OSA-18; ShOM = *Short Evaluation of Orofacial Myofunctional Protocol*

Em relação aos dados nutricionais, foi utilizado o critério que considera o Índice de Massa Corpórea (IMC = peso [kg]/estatura [m2]), sexo e idade. Os pontos de corte formam escores de IMC por idade em crianças (*escore*-Z)^(21)^, tendo sido observada obesidade em 6 crianças e sobrepeso em 3 (Tabela 1).

Na triagem miofuncional orofacial, foi possível analisar 7 dos 10 itens que compõem o protocolo ShOM por meio das gravações realizadas durante as consultas no ambulatório dos Distúrbios do Sono do Hospital das Clínicas da FMB -UNESP/Botucatu. Foram avaliados os seguintes itens: modo respiratório, competência labial, tônus labial, posição da língua, posição da língua na deglutição, oclusão dentária, Teste de Rosenthal, conforme descrito na tabela 2. A média do grupo foi de 5,42 (±1,4), sendo observada pontuação mínima = 4 e máxima = 7. Todos os indivíduos apresentaram alterações miofuncionais orofaciais. Quanto maior a pontuação alcançada, maior o comprometimento miofuncional orofacial (Tabelas 1 e 2).

**Tabela 2:** Resultados dos itens avaliados pelo protocolo ShOM

ShOM	SD (N=12)	
Pontuação máxima = 7	Alterado	Normal
Modo Respiratório	10 (83,33%)	2 (16,66%)
Competência de lábios	6 (50%)	6 (50%)
Tônus dos lábios	10 (83,33%)	2 (16,66%)
Posição da língua	12 (100%)	0
Posição da língua na deglutição	12 (100%)	0
Oclusão	5 (41,66%)	7 (58,33%)
Teste de Rosenthal	10 (83,33%)	2 (16,66%)

Legenda: ShOM = Short Evaluation of Orofacial Myofunctional Protocol; SD = Síndrome de Down; N= número de indivíduos

Quanto aos dados polissonográficos, o IAH médio encontrado foi de 12,5 eventos por hora de sono (±7,24), sendo que 1 criança (8,33%) foi diagnosticada com AOS Leve (1,1 – 5 e/h), 5 crianças (41,66%) com AOS Moderada (5,1 – 10 e/h), e 6 crianças (50%) com AOS Grave (> 10 e/h) (Tabela 3). Além das apneias obstrutivas e hipopneias, também foram observadas apneias centrais, em que o esforço respiratório está ausente ou diminuído, com valor médio de 2,36 eventos por hora de sono (± 2,09), podendo se relacionar a outras condições de saúde como as alterações cardiovasculares, comorbidades frequentes nesta população. A média do percentual de saturação de oxigênio (SPO_2_) mínima foi 84,3 (± 5,94), para SpO_2_ média foi 95,7 (±1,5), e percentual de tempo abaixo de 90% de SpO_2_ foi 1,71 (±3,02) (Tabela 3).

**Tabela 3:** Correlação entre ShOM e IAH, ShOM e hipopneias

Correlação de Spearman*																				
M ± DP			IDADE	IMC	IAH		AO	AC	AM	HIPO		%SPO_2_ MIN		%SPO_2_ MED		%SPO_2_ <90	PSQ		OSA-18	ShOM
IDADE	8.08 (± 2,75)	Rho de Spearman	—																	
		p-valor	—																	
IMC	22,1 (± 4,73)	Rho de Spearman	0.462	—																
		p-valor	0.13	—																
IAH	12.5 (± 7,24)	Rho de Spearman	-0.289	-0.319	—															
		p-valor	0.363	0.313	—															
AO	3.16 (± 4,06)	Rho de Spearman	-0.13	0.207	0.273		—													
		p-valor	0.687	0.519	0.391		—													
AC	2.36 (± 2,09)	Rho de Spearman	0.454	-0.118	-0.018		-0.333	—												
		p-valor	0.138	0.716	0.957		0.29	—												
AM	0,01 (± 0,034)	Rho de Spearman	-0.098	-0.065	0.259		0.194	-0.39	—											
		p-valor	0.762	0.841	0.416		0.545	0.21	—											
HIPO	7,17 (± 6,46)	Rho de Spearman	-0.44	-0.473	0.734	**	-0.189	-0.168	0.259	—										
		p-valor	0.152	0.121	0.007		0.557	0.601	0.416	—										
%SPO_2_ MIN	84,3 (±5,94)	Rho de Spearman	0.074	-0.218	-0.62	^*^	-0.235	0.081	-0.26	-0.277		—								
		p-valor	0.819	0.497	0.032		0.463	0.803	0.415	0.384		—								
%SPO_2_ MED	95,7 (± 1,5)	Rho de Spearman	0.097	-0.093	-0.306		0.064	0.321	-0.231	-0.192		0.767	**	—						
		p-valor	0.765	0.774	0.333		0.843	0.308	0.471	0.55		0.004		—						
%SPO<90	1,71 (± 3,02)	Rho de Spearman	-0.165	0.229	0.36		0.105	-0.235	0.303	0.189		-0.899	^***^	-0.9	***	—				
		p-valor	0.609	0.474	0.251		0.745	0.462	0.339	0.557		< .001		< .001		—				
PSQ	10,1 (± 4,94	Rho de Spearman	-0.355	0.132	0.396		0.396	-0.116	-0.163	0.263		-0.213		0.138		0.102	—			
		p-valor	0.257	0.683	0.202		0.202	0.719	0.614	0.409		0.507		0.67		0.752	—			
OSA-18	66.8 (± 28,3)	Rho de Spearman	-0.22	0	0.088		0.007	0.028	-0.519	0.207		0.049		0.244		-0.115	0.8	**	—	
		p-valor	0.491	1	0.787		0.983	0.931	0.084	0.519		0.879		0.444		0.723	0.002		—	
ShOM	5.42 (± 1,38)	Rho de Spearman	-0.291	-0.165	0.604	*	0.154	-0.369	0.203	0.75	^**^	-0.176		-0.061		0.019	0.064		-0.059	—
		p-valor	0.358	0.608	0.038		0.633	0.238	0.526	0.005		0.584		0.849		0.953	0.843		0.856	—


* p < .05;

** p < .01;

*** p < .001

Legenda: M = média; DP = Desvio Padrão; IMC = índice de massa corpórea; IAH = índice de apneia e hipopneia; AO = apneia obstrutiva; AC = apneia central; AM = apneia mista; HIPO = hipopneias; %SPO_2_ MIN = porcentagem de saturação de oxigênio mínima; %SPO_2_ MÉD = porcentagem de saturação de oxigênio média; %SPO_2_ <90 = porcentagem de tempo com saturação de oxigênio abaixo de 90; PSQ = *Pediatric Sleep Questionnaire*; OSA-18 = Protocolo OSA-18; ShOM = *Short Evaluation of Orofacial Myofunctional Protocol*

Nos questionários relacionados ao sono, a pontuação média para o PSQ foi de 10,1 (± 4,94) e OSA-18 foi de 66,8 (± 28,3). A pontuação alcançada nos dois questionários sugere risco aumentado para AOS (Tabela 3).

Após a aplicação do teste de coeficiente de correlação de *Spearman*, foi possível observar uma correlação positiva entre o ShOM e IAH (p=0,038), e entre o ShOM e número de hipopneias (p=0,005) conforme consta na Tabela 3. A correlação entre as variáveis descritas evidenciou que pontuações mais altas no ShOM se relacionam com maior gravidade da AOS.

## DISCUSSÃO

A AOS acomete entre 31% e 71% das crianças com SD^(4,10,11)^, enquanto na população pediátrica com desenvolvimento típico, a prevalência é de 1% a 4%^(7)^. O risco aumentado de AOS em crianças com SD se deve, especialmente, aos fenótipos relacionados à hipotonia e características craniofaciais como a hipoplasia de terço facial médio e base craniana.

Na SD, a condição muscular e as funções orofaciais e faríngeas contribuem para o colapso completo ou parcial da VAS durante as apneias e/ou hipopneias obstrutivas durante o sono, influenciando assim, no IAH, um dos parâmetros de gravidade da AOS^(22)^. Os resultados obtidos nesse estudo com crianças com SD e AOS evidenciaram a presença de alterações miofuncionais orofaciais em todas as crianças, com níveis variados de comprometimento das estruturas orais quanto à postura, tônus de lábios e língua e alterações nas funções de respiração, mastigação e deglutição. Apesar da reduzida casuística, foi verificada uma correlação positiva (p=0,038) entre a pontuação alcançada no protocolo de ShOM e o IAH, e entre o ShOM e o número de hipopneias (p=0,005), sugerindo assim, que quanto maior a pontuação no ShOM, ou seja, quanto maior o comprometimento miofuncional orofacial, maior a gravidade da AOS.

A identificação de marcadores clínicos com potencial de predição da AOS em crianças com SD facilitaria o diagnóstico precoce e tratamento, bem como a prevenção de morbidades. No entanto, até o momento, a literatura apresenta associação inconsistente ou frágil entre preditores clínicos e biomarcadores para a presença de AOS na referida população^(23)^.

No presente estudo foram considerados 7 dos 10 itens que compõem o protocolo ShOM original. Tal fato se deu pela dificuldade na compreensão dos comandos pelos indivíduos da amostra para execução dos 3 testes excluídos (tipo respiratório, tônus nasal e espelho de Glatzel, ou pela dificuldade de análise exclusiva por meio dos vídeos. Ressalta-se que este protocolo possibilita avaliar as alterações miofuncionais orofaciais frequentes na população pediátrica com alterações respiratórias obstrutivas do sono de forma rápida e padronizada. Entretanto, ainda não foi realizada sua validação, bem como não foi estabelecida uma pontuação de corte como parâmetro de identificação de AOS Novos estudos com maior casuística deverão mensurar o potencial e peso de cada item isoladamente.

A média do escore geral foi de 5,42 (±1,4), correspondendo a 77% da pontuação máxima de 7, sendo observada respiração predominantemente oral ou mista, alteração no tônus e competência labial, alteração na postura de língua em repouso e postura de língua na deglutição, assim como alteração na oclusão dentária e no Teste de Rosenthal. No estudo multicêntrico de Corrêa et al.^(13)^, crianças brasileiras e italianas típicas com AOS foram avaliadas por PSG e submetidas aos protocolos ShOM, OSA-18 e *Sleep clinical Record* (SCR), protocolo italiano que analisa parâmetros clínicos e sintomas para estimar o risco para a AOS. Foram identificadas alterações miofuncionais orofaciais semelhantes a este estudo piloto, como alterações do tônus labial, modo respiratório (oral), má oclusão dentária, alteração de postura de língua no repouso e na deglutição, incompetência dos lábios, alteração no teste de Rosenthal e alteração no teste de Glatzel. A média no ShOM foi de 5,64 ± 2,27, correspondente a 54% da pontuação máxima de 10. No entanto, uma vez que foram considerados os 10 itens originais do protocolo, as crianças típicas com AOS expressaram uma pontuação mais baixa do que as crianças com SD e AOS deste estudo, sugerindo um maior comprometimento miofuncional orofacial das crianças deste estudo, o que é compatível com a literatura por se tratar de crianças com SD^(4,6,11,23)^.

Estudos demonstram dificuldade ou não conseguem comprovar que fatores como idade, sexo, exposição ao cigarro, achados clínicos, dados antropométricos e presença de comorbidades, a não ser a doença cardíaca congestiva, sejam considerados preditivos da severidade da AOS em crianças com SD^(23,24)^. Entretanto, até o momento, nunca fora investigada e identificada a correlação apresentada nesse estudo piloto entre alterações nos aspectos miofuncionais orofaciais e a gravidade da AOS.

Quanto ao período de maior incidência da AOS na criança, estudos apontam a idade entre 2 e 8 anos de idade^(7)^. Diez et al.^(24)^ descrevem como fatores preditivos para a AOS, crianças com idade abaixo de oito anos, do sexo masculino e com hipertofria adenotonsilar. A idade média do grupo estudado foi de 8,08 anos (± 2,75), sendo 6 crianças do gênero masculino e 6 do feminino. 25% das crianças apresentaram sobrepeso e 50%, obesidade, mas sem correlação estatística com o IAH. Dyken et al.^(25)^ afirmam haver uma associação entre IMC mais elevado em crianças mais velhas e AOS severa. A correlação entre sobrepeso e obesidade já é comprovada na população adulta, no entanto, ainda há controvérsias quando se trata de crianças^(23)^. Há que se considerar que o grupo estudado possui outras características influenciadoras na gravidade do distúrbio além do IMC elevado, quais sejam a própria condição genética e comprometimento da morfologia e funcionalidade das estruturas orofaríngeas e do trato respiratório^(12,23)^. Em estudo conduzido por Hizal et al.^(24)^, não foram encontradas correlações entre AOS, idade, IMC e tamanho das tonsilas, assim como os achados deste estudo referente à idade e IMC^(25)^.

A AOS na SD, embora muito prevalente, ainda é subdiagnosticada e apresenta uma grande variedade de comorbidades como disfunções neurocognitivas e de aprendizagem, alterações comportamentais, cardiovasculares e metabólicas. O desenvolvimento das comorbidades pode estar associado não somente ao IAH e dessaturações de oxigênio, mas à presença de apneias centrais, como identificada nesse estudo, média de 2,36 eventos por hora de sono (±2,09), interferindo, assim, no crescimento e o desenvolvimento infantil, fato que reforça a importância do diagnóstico precoce^(24,25)^.

A Academia Americana de Pediatria recomenda a investigação da AOS na SD precocemente aos 6 meses, quando há queixas como respiração “pesada”, roncos, posição incomum ao dormir, sonolência diurna, paradas respiratórias e problemas comportamentais^(26)^. Na ausência de queixas durante as visitas pediátricas de rotina, é sugerida a realização da polissonografia (PSG) – exame padrão-ouro para diagnóstico de distúrbios do sono – entre 3 e 4 anos de idade para todas as crianças com SD^(11,24,26)^. Entretanto, mesmo em um ambiente acadêmico, como o local de desenvolvimento deste estudo, a acessibilidade da criança com SD à PSG ainda é restrito e mais tardio do que o recomendado. Este fato se relaciona ao elevado custo do exame, reduzida oferta de serviços com equipamentos e equipe especializada na realização da PSG na população pediátrica^(4,7,15,27)^.

Além da PSG, instrumentos de rastreio relacionados ao sono da criança como o PSQ^(15)^ e OSA-18^(16,17)^ fazem parte da rotina de investigação de indivíduos com transtornos do sono, auxiliando na identificação de risco para o diagnóstico da AOS. São ferramentas importantes na impossibilidade da realização da PSG. São considerados de fácil e rápida aplicação e buscam analisar a presença de sintomas, frequência, repercussões físicas e comportamentais. No entanto, a validação desses questionários na criança é limitada, uma vez que somente de 13% a 27% dos pais/responsáveis reconhecem a existência de problemas relacionados ao sono das suas crianças^(28)^.

O PSQ investiga diferentes aspectos da qualidade do sono, roncos e questões comportamentais como desatenção e hiperatividade. Possui sensibilidade e especificidade avaliada em 78% e 72%, respectivamente^(19)^, demonstrando que a avaliação da qualidade de sono pode ser um importante parâmetro na identificação de crianças com potencial de risco para AOS^(29)^. Nesse estudo, a pontuação média do PSQ foi de 9,6 (± 5,5), evidenciando risco aumentado para AOS nas crianças com SD, mas sem correlação com a gravidade da AOS.

O OSA-18 reflete o impacto do sono na qualidade de vida das crianças, considerando o sofrimento físico, sofrimento emocional, problemas diurnos e preocupação dos pais. No entanto, na amostra estudada, não foi identificada uma correlação entre a pontuação média de 66.8 (± 28.6), que significa impacto moderado na qualidade de vida, e o IAH, confirmando dados já descritos nos *guidelines* do *European Respiratory Society Task Force,* que sugerem que o questionário possui baixa sensibilidade e especificidade^(29,30)^. Também não foi verificada correlação entre as condições miofuncionais orofaciais e a pontuação obtida nesses instrumentos de rastreio do sono infantil.

No estudo de Corrêa et al.^(13)^, foi demonstrada uma correlação entre alteração na posição da língua em repouso e na deglutição com um maior IAH em crianças típicas com AOS, corroborando assim, com o resultado observado neste estudo piloto que evidenciou relação entre alterações posturais e funcionais das estruturas orofaríngeas e um maior número de apneia/hipopneias obstrutivas do sono.

Embora a reduzida casuística seja fator de ressalvas aos resultados apontados por esse estudo piloto, foi possível comprovar a hipótese que a identificação das condições miofuncionais orofaciais de crianças com SD pode auxiliar na identificação de risco e gravidade da AOS. Assim, considerando que os distúrbios miofuncionais orofaciais (DMOs) estão presentes em menor ou maior grau em indivíduos com SD, é imprescindível a identificação e quantificação das alterações da postura, mobilidade e tônus da musculatura orofacial, assim como nas funções de respiração, sucção, deglutição, mastigação e fala por meio da inserção do fonoaudiólogo nas equipes multidisciplinares de especialistas do sono^(4,6,11-13)^.

Esse é um estudo piloto de forma que mais pesquisas são necessárias contendo uma população determinada por um cálculo amostral a fim de compreender a relação entre as alterações miofuncionais orofaciais em crianças com SD e a presença e gravidade da AOS, bem como validar os questionários utilizados nessa população.

Por fim, a busca por instrumentos e métodos auxiliares no diagnóstico dos distúrbios respiratórios obstrutivos do sono na criança objetiva otimizar a identificação de risco e gravidade da AOS em situações de dificuldade e ou impossibilidade de acesso à PSG, exame padrão ouro para diagnóstico de distúrbios do sono. O diagnóstico assertivo e precoce possibilita minimizar comorbidades, identificando de maneira objetiva os padrões respiratórios obstrutivos e centrais durante o sono que impactam negativamente na saúde física, cognitiva e comportamental das crianças com SD e AOS.

## CONCLUSÕES

A avaliação da condição miofuncional orofacial pode ser considerada um recurso importante para aplicação clínica na investigação de risco para AOS em crianças com SD, sendo observada uma correlação positiva entre o ShOM e o IAH, e ShOM e número de hipopneias, ou seja, quanto maior a pontuação, e, portanto, pior condição miofuncional orofacial, maior a gravidade da AOS. No entanto, é possível inferir a importância em adotar a avaliação fonoaudiológica como parte integrante da investigação de risco para os distúrbios respiratórios do sono.
